# Supplementary material for: Microvascular immunity is organ-specific and remodeled after kidney injury in mice
Source: Nat Commun. 2025 May 9;16:4333. doi: 10.1038/s41467-025-59609-1 (PMC12064663; doi:10.1038/s41467-025-59609-1)
Supplement: Supplementary file 1 — Supplementary Information [file 41467_2025_59609_MOESM1_ESM.pdf]

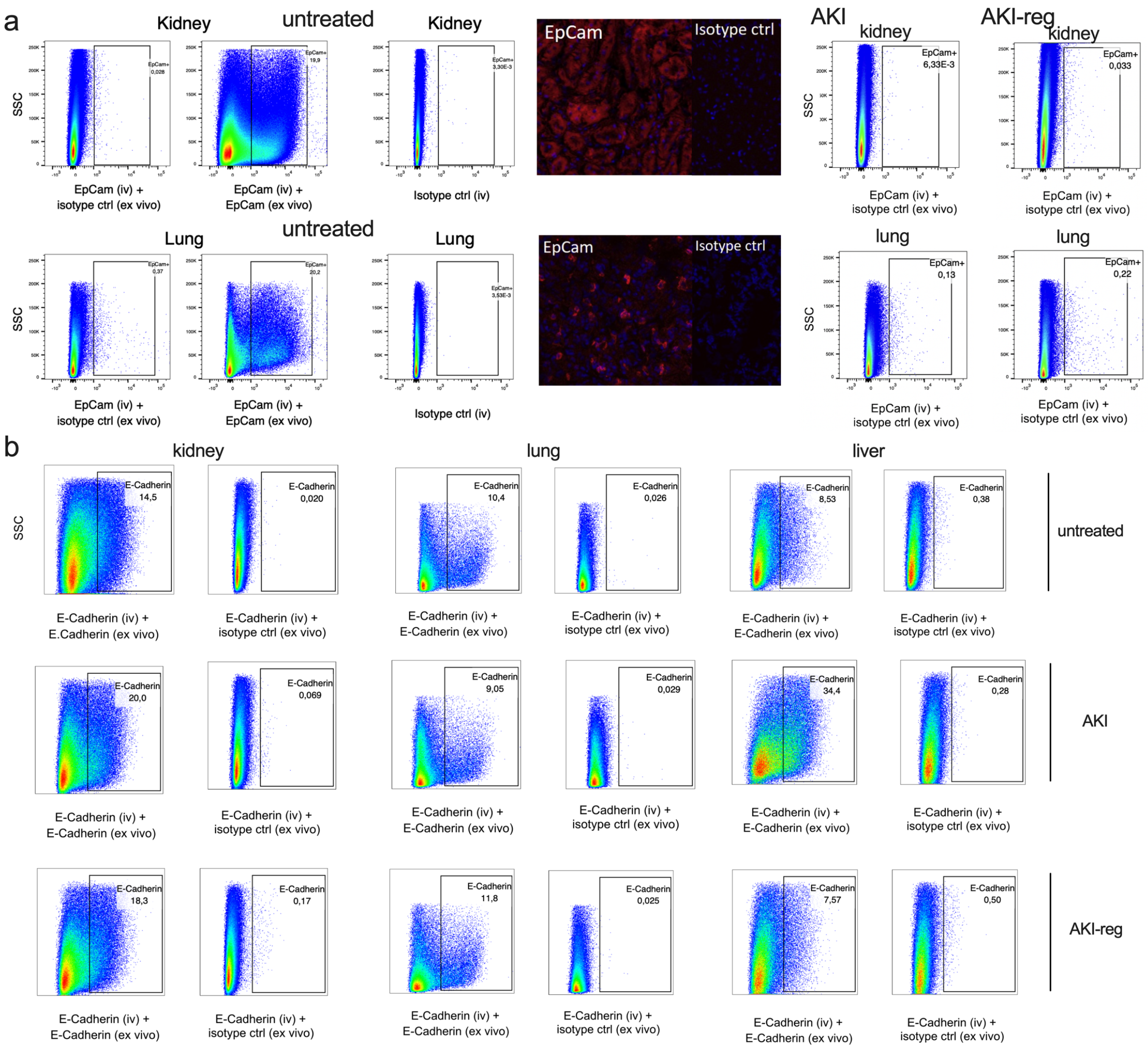

Supplemental figure 1: a) Intravenous injection of the anti-Epcam (a) and anti-E-cadherin (b) antibodies (directed at extravascular epithelial cells) does not stain significant cell numbers in kidney, liver and lung tissue of untreated, AKI- and AKI-reg treated mice. In contrast, the ex vivo staining detects about 15-20% positive cells, demonstrating that our experimental protocol specifically labels intravascular cells. Liver tissue is negative for Epcam. Representative plots of 3 independent experiments.

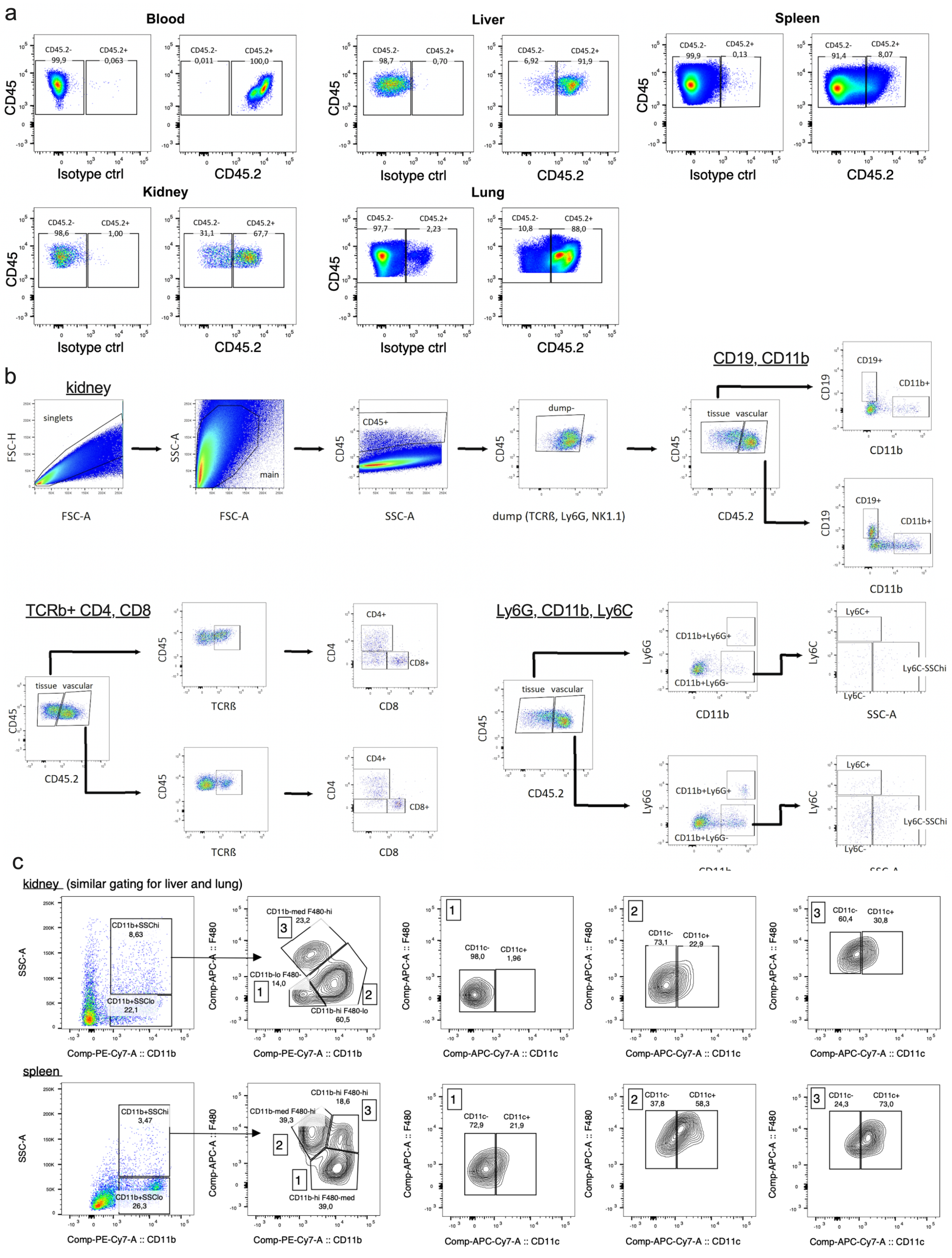

Supplemental figure 2: Flow cytometry gating strategies a) for intravascular (CD45.2+ CD45+) and extravascular (CD45.2- CD45+) leukocytes in different organs, b) for CD19+ B cells (top), TCRb+ CD4+ or CD8+ T cells (bottom left), CD11b+Ly6G+ neutrophils and three different types of monocytes (bottom right), and c) for CD11b+SSChigh cells using F480 and CD11c. Representative plots of 3 independent experiments.

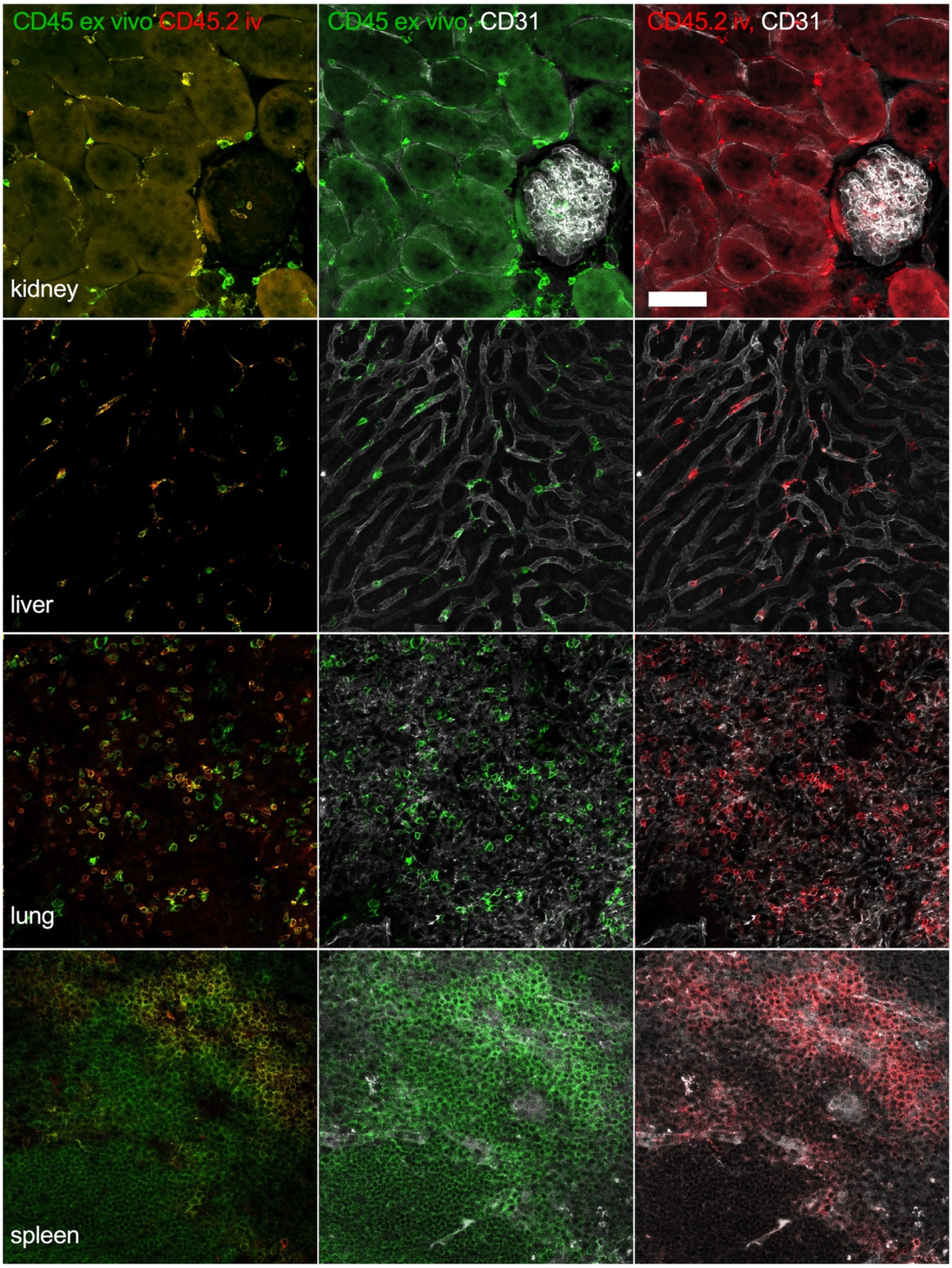

Supplemental figure 3: Tissue clearing and 3D confocal imaging of different organs stained with microvascular CD45.2 (red, iv injection), CD45 (green, ex vivo staining) and CD31 (white, iv injection). Representative plots for 4 biological replicates.

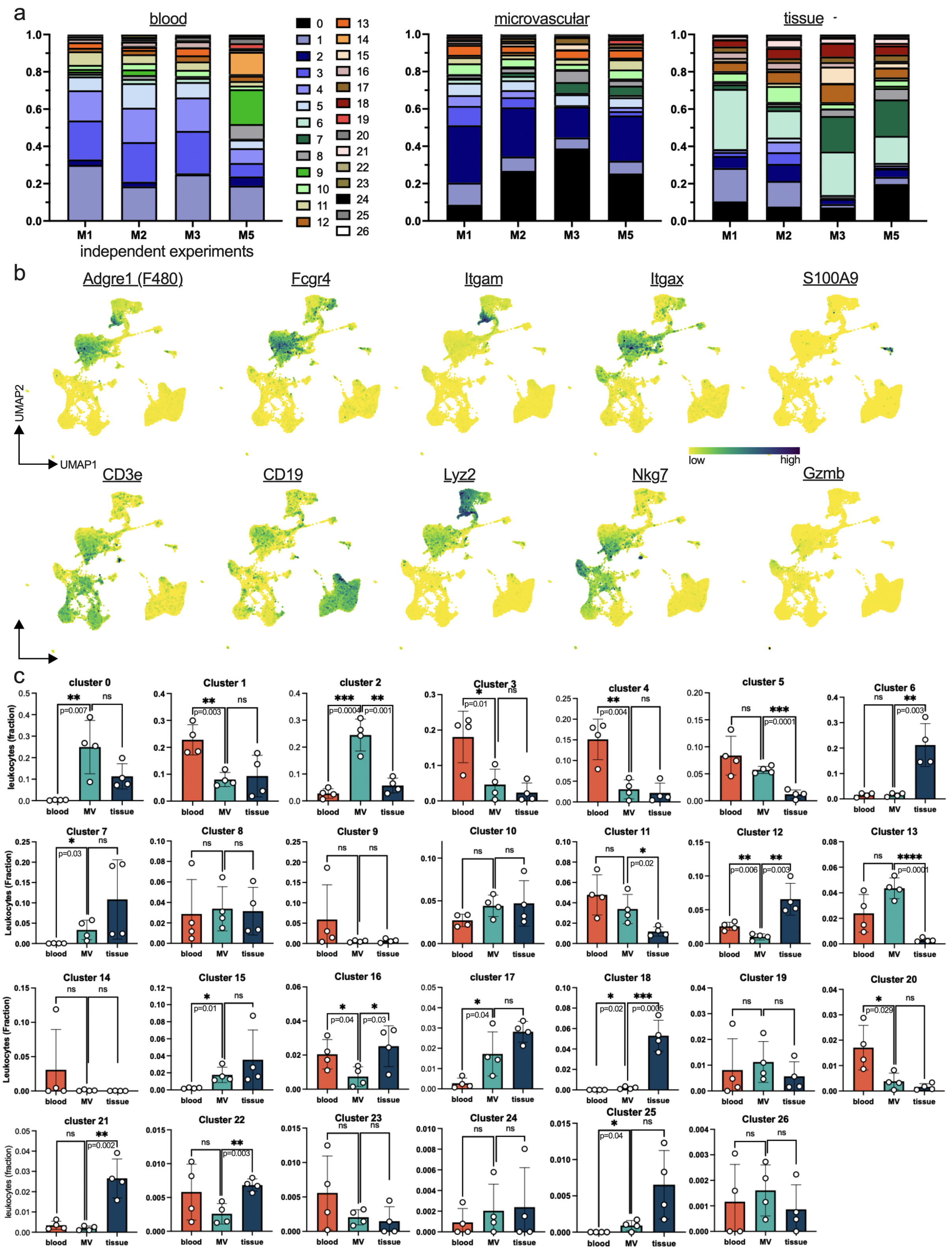

Supplemental figure 4: Figure corresponds to main figure 2. a) Cluster distribution of 26 cell clusters in each of the 4 untreated (UT) samples. b) Gene expression of selected genes shown on the UMAP plot. c) Leukocyte abundance for each of the 26 clusters by compartment. n=4 independent experiments. Mean and SD are shown. Paired two sided t test comparing the microvascular (MC) with the tissue or the blood compartment. * p < 0.05, ** p < 0.01

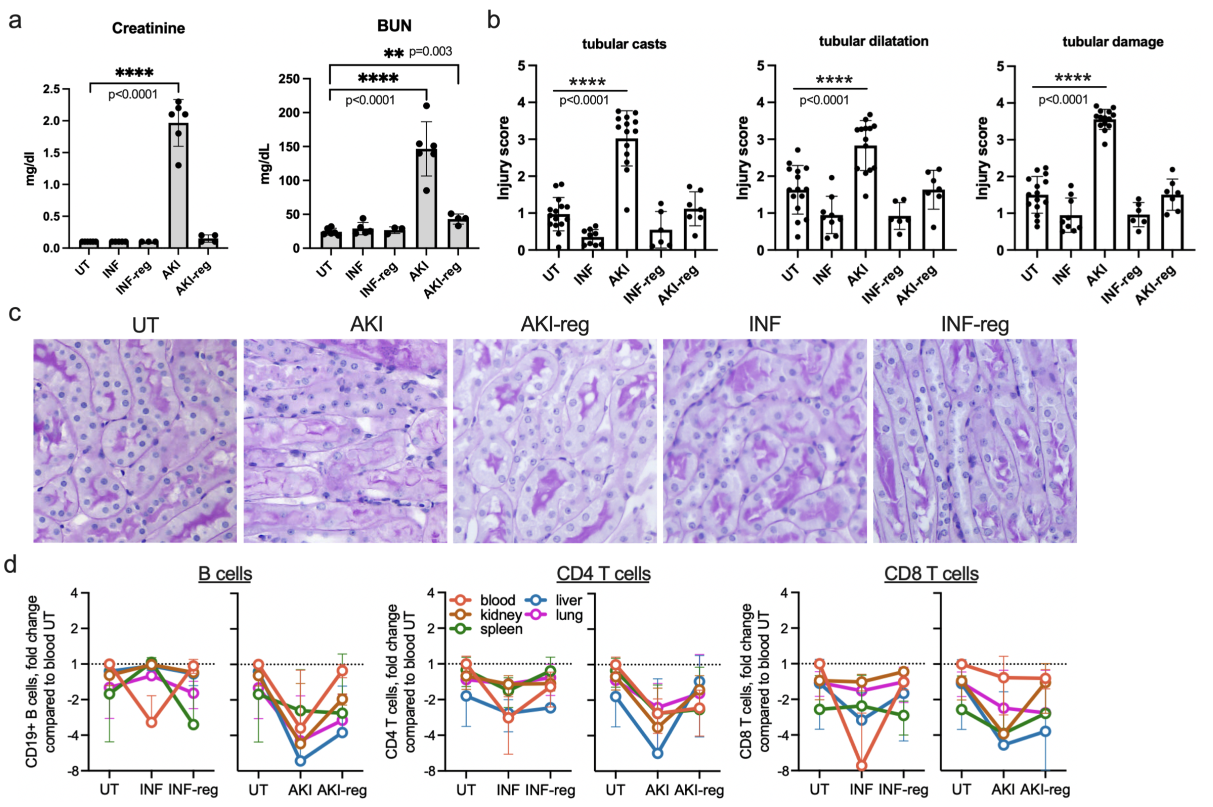

Supplemental figure 5: Figure corresponds to main figure 4. a,b) Every dot represents one biological replicate. Mean and SD are shown. Unpaired two sided t test comparing each condition with untreated (UT) ** $p < 0.01$, *** $p < 0.001$, **** $p < 0.0001$. a) Creatinine and blood urea nitrogen (BUN) measurements in blood serum in different disease models. b,c) Histological assessment of kidney injury in different disease conditions. Representative pictures of periodic-acid Schiff (PAS) stainings. d) Microvascular lymphocyte subtypes in different organs and the peripheral blood expressed as relative to blood untreated (equals 1). Mean and SD of n=4 independent experiments are shown.

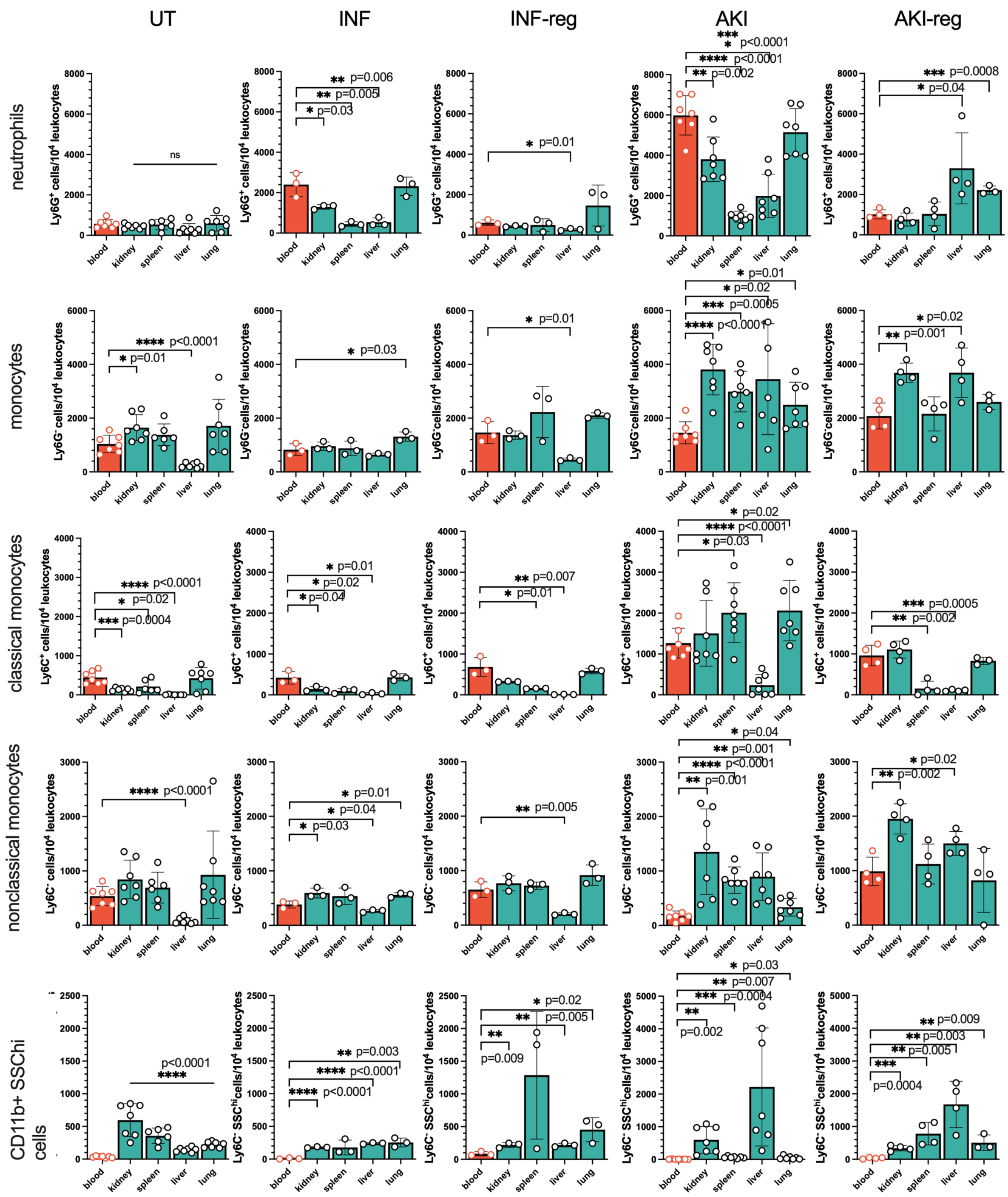

Supplemental figure 6: Figure corresponds to main figure 4. Flow cytometry analysis of microvascular leukocyte subtypes in comparison to peripheral blood (red bar). Every dot of one bar represents one biological replicate. Mean and SD are shown. Unpaired two sided t test of each organ compared to blood. * $p < 0.05$, ** $p < 0.01$, *** $p < 0.001$, **** $p < 0.0001$

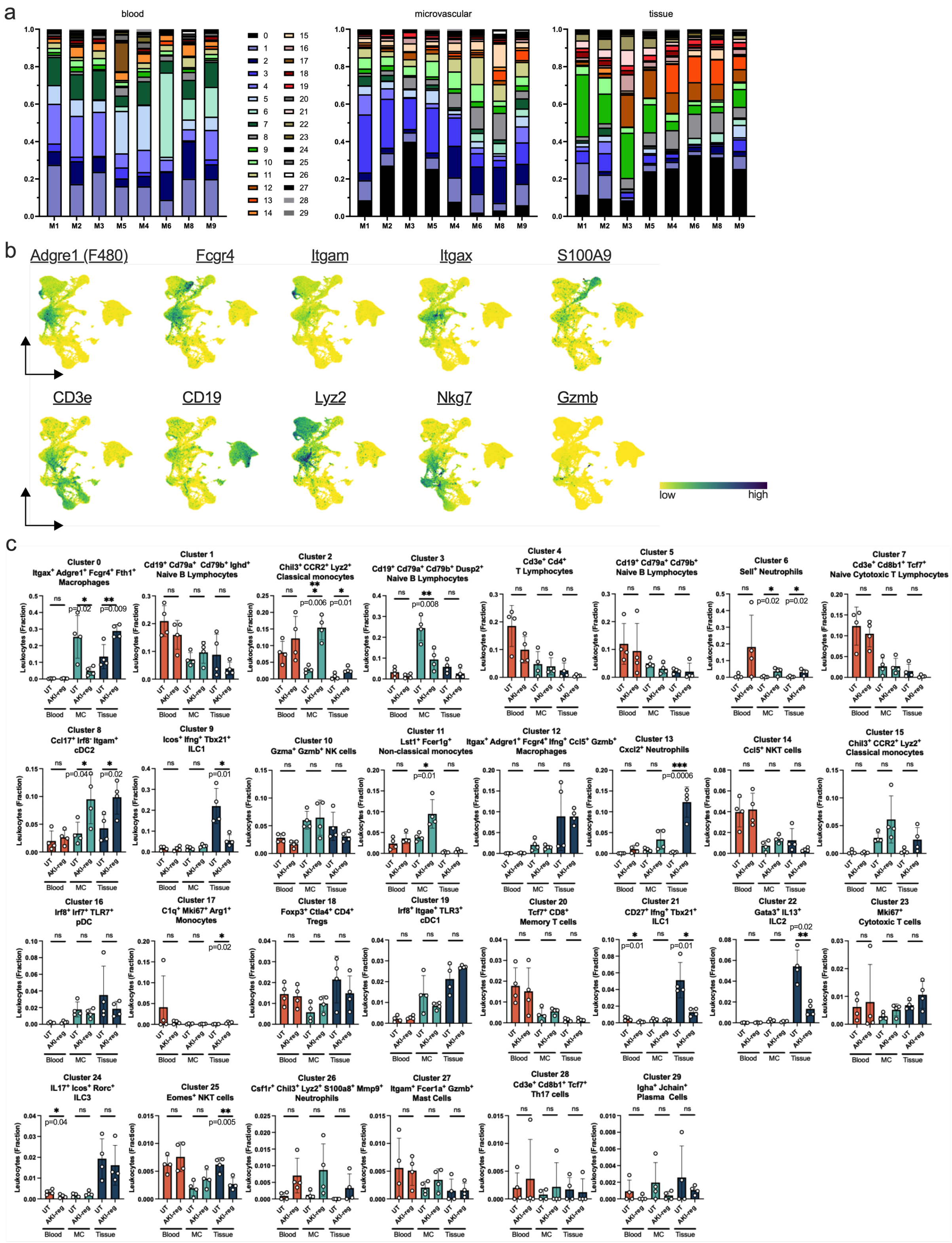

Supplemental figure 7: Figure corresponds to main figure 5. a) Cluster distribution of 29 cell clusters in each of the 8 samples which include 4 samples “untreated” (M1, M2, M3, M5; as shown in main figure 2) and 4 samples “AKI-reg” (M4, M6, M8, M9). b) Gene expression of selected genes shown on the UMAP plot. c) Leukocyte abundance for each of the 29 clusters by compartment. n=4 untreated and n=4 AKI-reg, independent experiments. Mean and SD are shown. The unpaired two sided t test was used to test significant differences between untreated (UT) and AKI-reg in the compartments blood, microcirculation (MC) and renal tissue. * p < 0.05, ** p < 0.01, *** p < 0.001

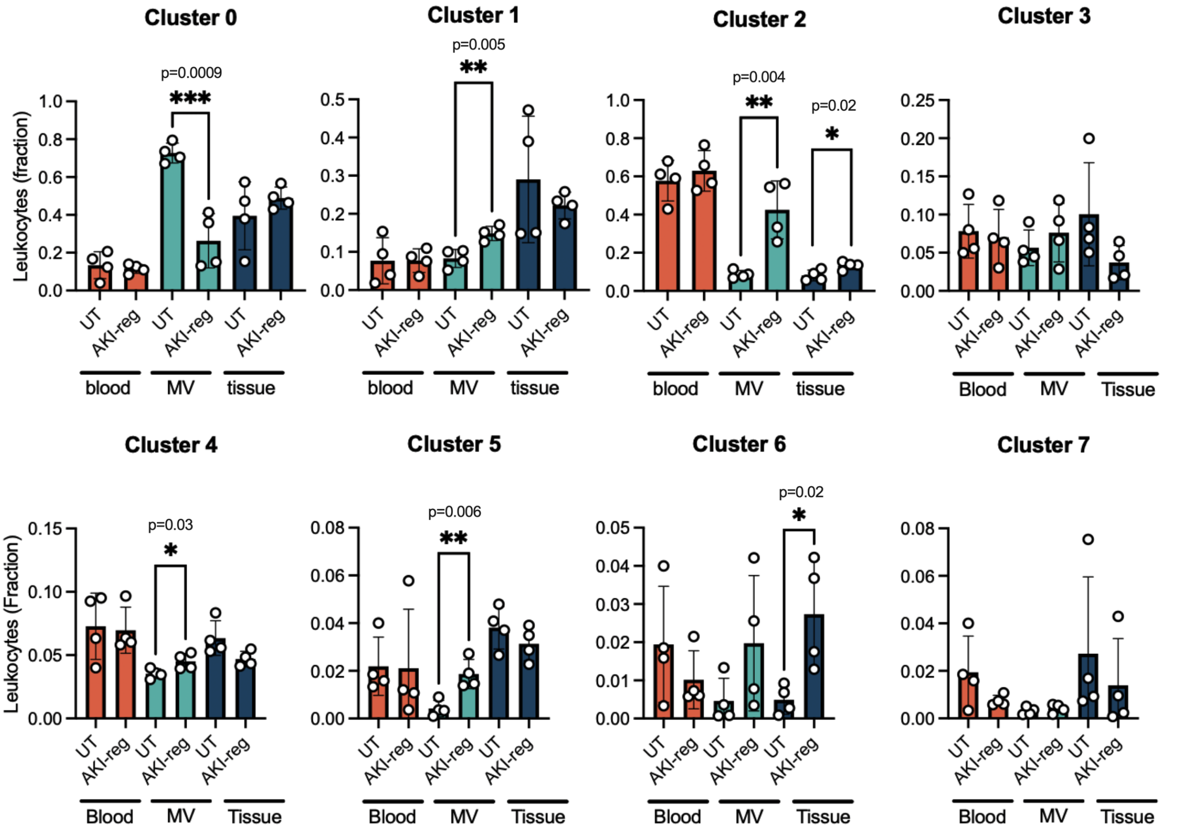

Supplemental figure 8: Subclustering of macrophages and DC. Refers to figure 7. MV = microvasculature. Each dot of one bar represents one biological replicate. Mean and SD are shown. Unpaired two sided t test between UT (untreated) and AKI-reg (regeneration after AKI). * $p < 0.05$, ** $p < 0.01$, *** $p < 0.001$
