# Supplementary material for: Microvascular immunity is organ-specific and remodeled after kidney injury in mice
Source: Nat Commun. 2025 May 9;16:4333. doi: 10.1038/s41467-025-59609-1 (PMC12064663; doi:10.1038/s41467-025-59609-1)
Supplement: Supplementary file 2 — Description of Additional Supplementary Files [file 41467_2025_59609_MOESM2_ESM.pdf]

Description of Additional Supplementary Files

File Name: Supplementary Data 1

Description: Cluster distribution (%) of all leukocytes in the blood, renal microvasculature (MV) and renal tissue. Related to figure 2 and 5.

File Name: Supplementary Data 2

Description: Cluster-specific differentially expressed genes (DEG) in all data sets. Related to figure 2, 3, 5, and 6.

File Name: Supplementary Data 3

Description: List of antibodies, reagents and the customized gene panel for sequencing.
