# Supplementary material for: Microvascular immunity is organ-specific and remodeled after kidney injury in mice
Source: Nat Commun. 2025 May 9;16:4333. doi: 10.1038/s41467-025-59609-1 (PMC12064663; doi:10.1038/s41467-025-59609-1)
Supplement: Supplementary file 6 — Reporting Summary [file 41467_2025_59609_MOESM6_ESM.pdf]

nature portfolio

Corresponding author(s): Buscher

Last updated by author(s): Jan 15, 2025

Reporting Summary

Nature Portfolio wishes to improve the reproducibility of the work that we publish. This form provides structure for consistency and transparency in reporting. For further information on Nature Portfolio policies, see our Editorial Policies and the Editorial Policy Checklist.

Statistics

For all statistical analyses, confirm that the following items are present in the figure legend, table legend, main text, or Methods section.

n/a	Confirmed	
☐	☒	The exact sample size (*n*) for each experimental group/condition, given as a discrete number and unit of measurement
☐	☒	A statement on whether measurements were taken from distinct samples or whether the same sample was measured repeatedly
☐	☒	The statistical test(s) used AND whether they are one- or two-sided<br>*Only common tests should be described solely by name; describe more complex techniques in the Methods section.*
☒	☐	A description of all covariates tested
☐	☒	A description of any assumptions or corrections, such as tests of normality and adjustment for multiple comparisons
☐	☒	A full description of the statistical parameters including central tendency (e.g. means) or other basic estimates (e.g. regression coefficient) AND variation (e.g. standard deviation) or associated estimates of uncertainty (e.g. confidence intervals)
☐	☒	For null hypothesis testing, the test statistic (e.g. *F*, *t*, *r*) with confidence intervals, effect sizes, degrees of freedom and *P* value noted<br>*Give P values as exact values whenever suitable.*
☒	☐	For Bayesian analysis, information on the choice of priors and Markov chain Monte Carlo settings
☒	☐	For hierarchical and complex designs, identification of the appropriate level for tests and full reporting of outcomes
☒	☐	Estimates of effect sizes (e.g. Cohen's *d*, Pearson's *r*), indicating how they were calculated

Our web collection on statistics for biologists contains articles on many of the points above.

Software and code

Policy information about availability of computer code

Data collection	FACSDiva software v8.0 (BD Biosciences)
Data analysis	FlowJo v10.9. software (BD Bioscience), ImageJ/Fiji v2.15 (http://fiji.sc/), Zen Software v3.8 (Zeiss, Germany), SevenBridges v1.11, R v4.0.5 and Seurat v4

For manuscripts utilizing custom algorithms or software that are central to the research but not yet described in published literature, software must be made available to editors and reviewers. We strongly encourage code deposition in a community repository (e.g. GitHub). See the Nature Portfolio guidelines for submitting code & software for further information.

Data

Policy information about availability of data

All manuscripts must include a data availability statement. This statement should provide the following information, where applicable:

- Accession codes, unique identifiers, or web links for publicly available datasets
- A description of any restrictions on data availability
- For clinical datasets or third party data, please ensure that the statement adheres to our policy

The sequencing data was uploaded in the repository GSE252496.

Research involving human participants, their data, or biological material

Policy information about studies with human participants or human data. See also policy information about sex, gender (identity/presentation), and sexual orientation and race, ethnicity and racism.

Reporting on sex and gender	*Use the terms sex (biological attribute) and gender (shaped by social and cultural circumstances) carefully in order to avoid confusing both terms. Indicate if findings apply to only one sex or gender; describe whether sex and gender were considered in study design; whether sex and/or gender was determined based on self-reporting or assigned and methods used. Provide in the source data disaggregated sex and gender data, where this information has been collected, and if consent has been obtained for sharing of individual-level data; provide overall numbers in this Reporting Summary. Please state if this information has not been collected. Report sex- and gender-based analyses where performed, justify reasons for lack of sex- and gender-based analysis.*
Reporting on race, ethnicity, or other socially relevant groupings	*Please specify the socially constructed or socially relevant categorization variable(s) used in your manuscript and explain why they were used. Please note that such variables should not be used as proxies for other socially constructed/relevant variables (for example, race or ethnicity should not be used as a proxy for socioeconomic status). Provide clear definitions of the relevant terms used, how they were provided (by the participants/respondents, the researchers, or third parties), and the method(s) used to classify people into the different categories (e.g. self-report, census or administrative data, social media data, etc.) Please provide details about how you controlled for confounding variables in your analyses.*
Population characteristics	*Describe the covariate-relevant population characteristics of the human research participants (e.g. age, genotypic information, past and current diagnosis and treatment categories). If you filled out the behavioural & social sciences study design questions and have nothing to add here, write "See above."*
Recruitment	*Describe how participants were recruited. Outline any potential self-selection bias or other biases that may be present and how these are likely to impact results.*
Ethics oversight	*Identify the organization(s) that approved the study protocol.*

Note that full information on the approval of the study protocol must also be provided in the manuscript.

Field-specific reporting

Please select the one below that is the best fit for your research. If you are not sure, read the appropriate sections before making your selection.

☒ Life sciences ☐ Behavioural & social sciences ☐ Ecological, evolutionary & environmental sciences

For a reference copy of the document with all sections, see nature.com/documents/nr-reporting-summary-flat.pdf

Life sciences study design

All studies must disclose on these points even when the disclosure is negative.

Sample size	No sample size calculation was performed. We used 4 mice for sequencing experiments. The sample sufficed to identify significant differences between the blood, microvasculature and tissue.
Data exclusions	No data was excluded
Replication	All data could be replicated successfully using independent experiments. The number of independent experiments is indicated in the figure legend.
Randomization	Not applicable because wild type mice were used
Blinding	The investigators were not blinded during data collection and acquisition.

Reporting for specific materials, systems and methods

We require information from authors about some types of materials, experimental systems and methods used in many studies. Here, indicate whether each material, system or method listed is relevant to your study. If you are not sure if a list item applies to your research, read the appropriate section before selecting a response.

Materials & experimental systems

n/a	Involved in the study
☐	☒ Antibodies
☒	☐ Eukaryotic cell lines
☒	☐ Palaeontology and archaeology
☐	☒ Animals and other organisms
☒	☐ Clinical data
☒	☐ Dual use research of concern
☒	☐ Plants

Methods

n/a	Involved in the study
☒	☐ ChIP-seq
☐	☒ Flow cytometry
☒	☐ MRI-based neuroimaging

Antibodies

Antibodies used: All antibodies are listed in supplemental figure 1.

Validation: All primary antibodies are common in the field and had been extensively validated by the manufactures' as detailed online.

Animals and other research organisms

Policy information about studies involving animals; ARRIVE guidelines recommended for reporting animal research, and Sex and Gender in Research

Laboratory animals: C57BL/6 mice were purchased by Charles River, age 8-20 weeks

Wild animals: not applicabale

Reporting on sex: Only male mice were used because there is a sex-specific difference to the disease model AKI (see methods)

Field-collected samples: not applicable

Ethics oversight: All animal experiments were approved by the authorities (Landesamt für Natur, Umwelt und Verbraucherschutz, 81-02.04.2019.A253) and performed in accordance with the animal protection guidelines of Germany.

Note that full information on the approval of the study protocol must also be provided in the manuscript.

Plants

Seed stocks: *Report on the source of all seed stocks or other plant material used. If applicable, state the seed stock centre and catalogue number. If plant specimens were collected from the field, describe the collection location, date and sampling procedures.*

Novel plant genotypes: *Describe the methods by which all novel plant genotypes were produced. This includes those generated by transgenic approaches, gene editing, chemical/radiation-based mutagenesis and hybridization. For transgenic lines, describe the transformation method, the number of independent lines analyzed and the generation upon which experiments were performed. For gene-edited lines, describe the editor used, the endogenous sequence targeted for editing, the targeting guide RNA sequence (if applicable) and how the editor was applied.*

Authentication: *Describe any authentication procedures for each seed stock used or novel genotype generated. Describe any experiments used to assess the effect of a mutation and, where applicable, how potential secondary effects (e.g. second site T-DNA insertions, mosiacism, off-target gene editing) were examined.*

Flow Cytometry

Plots

Confirm that:

☒ The axis labels state the marker and fluorochrome used (e.g. CD4-FITC).

☒ The axis scales are clearly visible. Include numbers along axes only for bottom left plot of group (a 'group' is an analysis of identical markers).

☒ All plots are contour plots with outliers or pseudocolor plots.

☒ A numerical value for number of cells or percentage (with statistics) is provided.

Methodology

Sample preparation: Heparinized blood was incubated with Fc Block (Biolegend) for 10 min on ice followed by antibody staining for 30 min. Erythrocytes (RBC) were lysed for 10 min at room temperature (RT) using 1x RBC Lysis/Fixation solution (Biolegend). Samples

were washed with phosphate buffered saline (PBS) at 400x g for 5 min and cell pellets were resuspended in PBS with 1% fetal calf serum (FCS). Organs were minced into small pieces, and kidney, liver and lung were digested in 1 mL HBSS (with Ca/Mg) including DNAse I (10mg/mL) and collagenase I (100 U/µL) at 37°C and 350 rpm for 30 min (kidney) or 45 min (liver and lung). 2 ml PBS was added to the digestion buffer followed by filtering through a 70 µm cell strainer. Samples were then centrifuged at 400g for 5 min and cell pellets were resuspended in PBS with 1% FCS. Cells were incubated with antibody mixtures (1:100) for 30 min on ice, followed by fixation with 4% PFA for 10 min. RBC lysis was also required for the spleen tissue. Lineage markers were used for negative gating (Lin-). This included the markers (Ly6G, CD11b) and (CD19, NK1.1, TCR) for lymphoid and myeloid panels, respectively. Cells were washed with 2 ml PBS (400g, 5 min) and resuspended in PBS with 1% FCS.

Instrument	All samples were measured using BD FACSCanto II (BD Biosciences).
Software	All samples were acquired using the FACSDiva software 8.0 (BD Biosciences). Data analysis was performed using FlowJo v10.9. software (BD Bioscience).
Cell population abundance	The sorted populations were > 95% pure as determined by sorting experiments with a post-sort purity control by flow cytometry.
Gating strategy	See supplemental figure 2

☒ Tick this box to confirm that a figure exemplifying the gating strategy is provided in the Supplementary Information.
